# Niobium-substituted octahedral molecular sieve (OMS-2) materials in selective oxidation of methanol to dimethoxymethane†

**DOI:** 10.1039/c9ra04804a

**Published:** 2019-10-14

**Authors:** Niluka D. Wasalathanthri, Curtis Guild, Quddus A. Nizami, Shanka L. Dissanayake, Junkai He, Peter Kerns, Jared Fee, Laura Achola, Dinithi Rathnayake, Chandima Weerakkody, Steven L. Suib, Partha Nandi

**Affiliations:** Department of Chemistry, University of Connecticut Storrs Connecticut 06269 USA steven.suib@uconn.edu; Institute of Material Science, University of Connecticut Storrs Connecticut 06269 USA; Corporate Strategic Research, ExxonMobil Research and Engineering Company Annandale New Jersey 08801 USA partha.nandi@exxonmobil.com

## Abstract

Octahedral molecular sieve (OMS-2) refers to a one-dimensional 2 × 2 framework of octahedral manganese oxo units based on the cryptomelane-type framework. Herein, we describe a niobium (Nb) substituted mixed metal oxide of Nb and Mn where the cryptomelane-type framework is retained. These materials are hydrothermally synthesized from the reaction of potassium permanganate, manganese sulfate, and homogeneous niobium(v) precursors. Niobium incorporation up to 31 mol% can be achieved without destroying the one dimensional 2 × 2 framework. The yields of the materials vary between 70 and 90%. These materials are analyzed by powder XRD, BET isotherm, TEM, SEM, XRF, and XPS studies. The synthesized materials show promising activity in selective oxidation of methanol to dimethoxymethane (DMM) at 200 °C. Normalized activity correlations followed the trend 21% Nb-OMS-2 > 15% Nb-OMS-2 > 31% Nb-OMS-2 > 68% Nb-OMS-2 > K-OMS-2. A fluctuation in methanol conversion was observed around 125–150 °C in most samples, suggesting this to be a catalytically important temperature regime when forming active sites for DMM production.

## Abstract 2

The prepared bifunctional Nb-OMS-2 catalysts are promising candidate in partial oxidation of methanol into dimethoxymethane at a lower temperature suggesting that there is an important temperature regime when forming active sites for DMM production.

## Graphical abstract

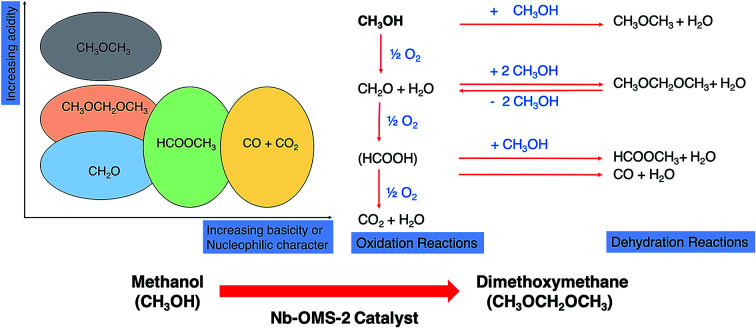

## Introduction

Octahedral molecular sieve (K-OMS-2) based on the cryptomelane framework^1–5^ has emerged as an important class of redox active heterogeneous catalysts for a multitude of catalytic partial oxidations.^6–9^ Prior work has shown that a fraction of Mn in these frameworks can be substituted with other metals and the degree of substitutions can vary quite significantly.^1–5^ Metal substitution in the K-OMS-2 framework impacts physical and chemical properties such as: aspect ratio of the rod (L/D), degree of aggregation, thermal stability, redox properties (*e.g.*, ratio of Mn^3+^/Mn^4+^), conductivity, and catalytic activity. In most cases the extent of Mn substitution is less than 5 wt%.^1^ However, in the case of V and Co the extent of substitution can be more than 10% where the OMS-2 structure is still retained. Unlike with V, the mixed metal oxides with Nb and Mn are known to exist in molecular crystals as [MnNb_9_O_28_]^8−^ or [H_2_MnNb_10_O_32_]^8−^ made from homogeneous precursors involving syntheses with organic templates.^10,11^ We studied whether it is possible to substitute Mn with relatively larger in size Nb in the octahedral molecular sieve framework, and if so to what extent in order to maintain the one dimensional 2 × 2 framework. In the present manuscript, we report Nb substituted OMS-2 materials where the extent of Mn substitutions by Nb can go up to 31%. Beyond this high level of substitution, these materials do not retain the cryptomelane structure and an amorphous phase of Nb_2_O_5_ starts to appear.

Dimethoxymethane (DMM; CH_3_OCH_2_OCH_3_) is an important chemical used in many industries as a building block in organic synthesis, a gasoline additive, and as precursor for concentrated formaldehyde (HCHO) streams.^12–15^ Furthermore, oxymethylene dimethyl ethers (OME) such as DMM are demonstrated as potential candidates for synthetic fuels in the recent times.^16^ These compounds can be used as substitutes to diesel or mixed into it. DMM is benign to the environment due to its high oxygen content and the absence of C–C bonds.^17^ As a result, the issue of diesel exhaust which has adverse effects on human health and ecosystem can be addressed.^18,19^

DMM synthesis can be achieved by partial oxidation of methanol to formaldehyde followed by nucleophilic addition of methanol to formaldehyde (twice).^12^ The overall balanced chemical equation for the reaction is given in eqn (E 1).^12,20^E 1
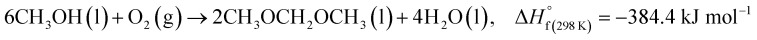


According to the literature, methanol oxidation may produce various products [formaldehyde (CH_2_O), dimethyl ether (CH_3_OCH_3_), methyl formate (HCOOCH_3_), dimethoxymethane (CH_3_OCH_2_OCH_3_), and carbon oxides (CO & CO_2_)] depending on various parameters such as catalyst, reaction temperature, reactant partial pressure, residence time, and methanol to oxygen ratio.^20^ The following reactions show different possible pathways for methanol oxidation.

E 2 2CH_3_OH(g) → CH_3_OCH_3_(g) + H_2_O(g) (dehydration)

E 3 CH_3_OH(g) + ½O_2_(g) → CH_2_O(g) + H_2_O(g) (oxidation)

E 4 CH_2_O(g) + 2CH_3_OH(g) → CH_3_OCH_2_OCH_3_(g) + H_2_O(g) (dehydration)

E 5 CH_2_O(g) + ½O_2_(g) → HCOOH*(g) + CH_3_OH(g) → HCOOCH_3_(g) + H_2_O(g) (oxidation + dehydration)

E 6 CH_2_O(g) + ½O_2_(g) → HCOOH*(g) → CO(g) + H_2_O(g) (oxidation + dehydration)

E 7 HCOOH*(g) + ½O_2_(g) → CO_2_(g) + H_2_O(g) (oxidation)

The partial oxidation of methanol to formaldehyde is a large-scale industrial process carried out over silver or copper catalysts.^21^ Due to its economic value, this reaction has been studied extensively in order to improve the efficiency of the process and to understand the reaction mechanism. Mixed and doped metal oxides have shown better activity towards the methanol partial oxidation reaction due to the variation in acidity and redox activity of the material.^22^ Acidic sites of the catalyst could drive the dehydration reaction to completely yield dimethyl ether. On the other hand, increase in the basic character of the catalyst will give formate species, which further decompose to carbon dioxide. For example, metal oxides with Lewis acidic sites, niobia, and alumina have been used to produce dimethyl ether from methanol. Metal oxides with redox sites such as vanadia, tin oxide, and molybdena have been studied as catalysts for methanol partial oxidation to produce formaldehyde and methyl formate.

Notably, vanadium-based systems show exceptional activity for the selective oxidation of methanol with most of the catalysts being supported catalysts and for some nanostructuring was key to the reactivity.^23,24^ The doping of SO_4_^2−^ and PO_4_^3−^ ions further improved the conversion and selectivity of the catalyst for the reaction. It has been suggested that acidic sites favor the condensation of formaldehyde to DMM, thus the addition of acidic additives has been vastly employed to improve the DMM yield.^25,26^ Others have co-doping vanadium with sulfur or phosphorus and still observe high activity.^12,27^ Gornay *et al.* reported a mixed metal oxide catalyst, FeMo (MoO_3_–Fe_2_(MoO_4_)_3_), with a high yield of DMM under high methanol partial pressures.^28^ Industrial scale production of DMM is currently carried out through a two-step process. First, oxidation of gas phase methanol to formaldehyde followed by a liquid phase acetylation step. However, synthesizing DMM *via* a direct single-step process is more economical due to minimized capital expenditure.^14^ The redox catalyzed pathways lead to a sequence of oxidized species whereas the acid-catalyzed pathways yield dehydration products. In this respect, the motivation for the work reported here is to develop a catalyst which oxidizes methanol to DMM with high selectivity. An appropriate system would be a bifunctional catalyst which contains adequate amounts of both acid and redox sites.^13,14^ Thus, incorporating redox innocent but acidic Nb into redox active K-OMS-2 would create a bifunctional mixed metal oxide catalytic systems for aerobically oxidizing methanol into DMM at lower temperature for potential energy and CO_2_ savings.

## Experimental

### Materials synthesis

Nb incorporation in OMS-2 framework was achieved by reacting soluble Nb precursors such as Nb(OEt)_5_ and Nb(C_2_O_4_H)_5_·*X*H_2_O with KMnO_4_ and MnSO_4_ in acidic medium. Other Nb sources such as niobic acid, niobium oxides, and LiNbO_2_ did not incorporate Nb in the OMS-2 framework in our experience. The syntheses of various Nb incorporated OMS-2 materials is described below:

#### 15 mol% Nb

In a 300 mL round bottom flask, a solution of MnSO_4_·H_2_O (Sigma Aldrich 99+%), was prepared by mixing solid manganese sulfate (25.35 g, 150 mmol); deionized water, 90 mL; and HNO_3_ (J.T. Baker 69–70%), 9 mL. In a beaker, a solution of KMnO_4_ (Sigma Aldrich 99+%), was prepared by mixing solid potassium permanganate (17.54 g, 111 mmol) and deionized water, 300 mL. Potassium permanganate solution was added dropwise to manganese sulfate solution while stirring the solution. Nb(OEt)_5_ (Sigma Aldrich 99.95%) (3.85 g, 12 mmol) was added to the solution. The mass of metal dopant was varied depending on the desired ratios of niobium to manganese. The resulting solution after being mixed was refluxed at 100–110 °C overnight. Upon cooling, the solution was filtered and washed with deionized water, and then dried to 120 °C overnight. After drying, the yield of the material was 12.73 g using between 25.35 and 17.54 g of manganese reactant.

#### 21 mol% Nb

In a 300 mL round bottom flask, a solution of MnSO_4_·H_2_O (Sigma Aldrich 99+%), was prepared by mixing solid manganese sulfate (25.35 g, 150 mmol); deionized water, 90 mL; and HNO_3_ (J.T. Baker 69–70%), 9 mL. In a beaker, a solution of KMnO_4_ (Sigma Aldrich 99+%), was prepared by mixing solid potassium permanganate (17.54 g, 111 mmol) and deionized water, 300 mL. Potassium permanganate solution was added dropwise to manganese sulfate solution while stirring the solution. Nb(OEt)_5_ (Sigma Aldrich 99.95%) (3.85 g, 12 mmol) was added to the solution. The mass of metal dopant was varied depending on the desired ratios of niobium to manganese. The resulting solution after being mixed was refluxed at 100–110 °C overnight. Upon cooling, the solution was filtered and washed with deionized water, and then dried at 120 °C overnight. After drying, the yield of the material was 14.74 g using between 25.35 and 17.54 g of manganese reactant.

#### 31 mol% Nb

In a 300 mL round bottom flask, a solution of MnSO_4_·H_2_O (Sigma Aldrich 99+ %), was prepared by mixing solid manganese sulfate (25.35 g, 150 mmol); deionized water, 90 mL; and HNO_3_ (J.T. Baker 69–70%), 9 mL. In a beaker, a solution of KMnO_4_ (Sigma Aldrich 99+%), was prepared by mixing solid potassium permanganate (17.54 g, 111 mmol) and deionized water, 300 mL. Potassium permanganate solution was added dropwise to manganese sulfate solution while stirring the solution. Nb(OEt)_5_ (Sigma Aldrich 99.95%) (11.45 g, 36 mmol) was added to the solution. The mass of metal dopant was varied depending on the desired ratios of niobium to manganese. The resulting solution after being mixed was refluxed at 100–110 °C overnight. Upon cooling, the solution was filtered and washed with deionized water, and then dried to 120 °C overnight. After drying, the yield of the material was 10.26 g using between 25.35 and 17.54 g of manganese reactant. XRF analysis confirmed the loading of Nb.

#### 68 mol% Nb

In a 300 mL round bottom flask, a solution of MnSO_4_·H_2_O (Sigma Aldrich 99+%), was prepared by mixing solid manganese sulfate (17.6 g, 104 mmol); deionized water, 90 mL; and HNO_3_ (J.T. Baker 69–70%), 6 mL. In a beaker, a solution of KMnO_4_ (Sigma Aldrich 99+%), was prepared by mixing solid potassium permanganate (9.5 g, 60 mmol) and deionized water, 200 mL. Potassium permanganate solution was added dropwise to manganese sulfate solution while stirring the solution. Nb(OEt)_5_ (Sigma Aldrich 99.95%) (15.26 g, 48 mmol) was added to the solution. The mass of metal dopant was varied depending on the desired ratios of niobium to manganese. The resulting solution after being mixed was refluxed at 100–110 °C overnight. Upon cooling, the solution was filtered and washed with deionized water, and then dried to 120 °C overnight. After drying, the yield of the material was 10.22 g using between 17.6 and 9.5 g of manganese reactant.

### Catalytic activity

Methanol partial oxidation reactions were carried out in a fixed-bed quartz reactor (i.d. 6 mm) containing 0.100 g of catalyst supported by quartz wool, operating at atmospheric pressure. A mixture of gas containing oxygen and helium were bubbled through methanol at room temperature (RT). The outlet gas was monitored by an on-line GC equipped with two columns (Hayesep and 13X siloxane) connected in parallel, and two detectors (thermal conductivity detector (TCD) and Helium ionization detector (HID)). The temperature at which maximum conversion of desired partial oxidation product (dimethoxy methane, DMM), was first investigated by feeding the system with 5% O_2_ balanced with He through a methanol bubbler at room temperature (RT). The flow rate was 50 standard cubic centimeters (sccm). Final methanol/oxygen/helium composition of the feed gas was approximately 13/4/83 mol%. Temperature was increased by 25 °C increments from (100–250) °C. Three injections were done at each temperature and the average activity is reported.

## Results and discussion

### Characterization

#### Elemental analyses (wt%)

The elemental composition of the materials was performed by X-ray Fluorescence technique. The elemental composition of the materials was estimated using a Rigaku ZSX Primus IV wavelength-dispersive XRF spectrometer with a tube above geometry and an ultra-thin end window having an X-ray tube with a 4 kW Rh anode.

Table 1 above shows the distribution of Mn, Nb and K by weight. Mn/K ratio gradually decreases with increasing Nb loading confirming the removal of potassium ions from the tunnel structure.^29,30^ For all the materials up to 31% Nb loading, the Nb/K ratio has increased further confirming the partial substitution of potassium ions. However, upon 68% loading of Nb, mass percentages of both Mn and K has drastically dropped compared to the materials with lower Nb loading. The results further proves that after 31% loading of Nb, the cryptomelane structure has collapsed and a material with dominating amount of Nb has formed.

**Table 1:** XRF composition of elements (by weight)

Material description	Mn (wt%)	Nb (wt%)	K (wt%)
15% Nb-OMS-2	75	16	9
21% Nb-OMS-2	71	19	10
31% Nb-OMS-2	48	43	9
68% Nb-OMS-2	16	80	4

#### Powder XRD analysis

**Fig. 1:**
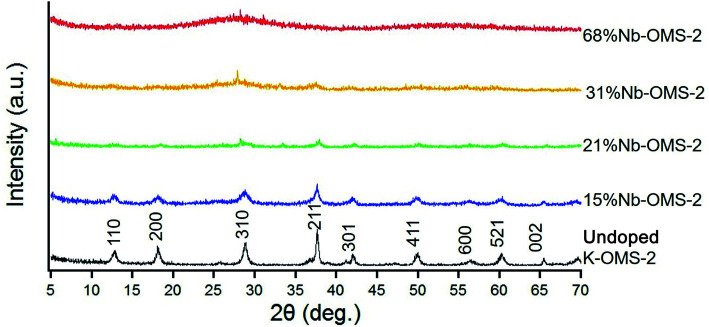
Powder XRD stack plot of Nb-OMS-2.

**Fig. 2:**
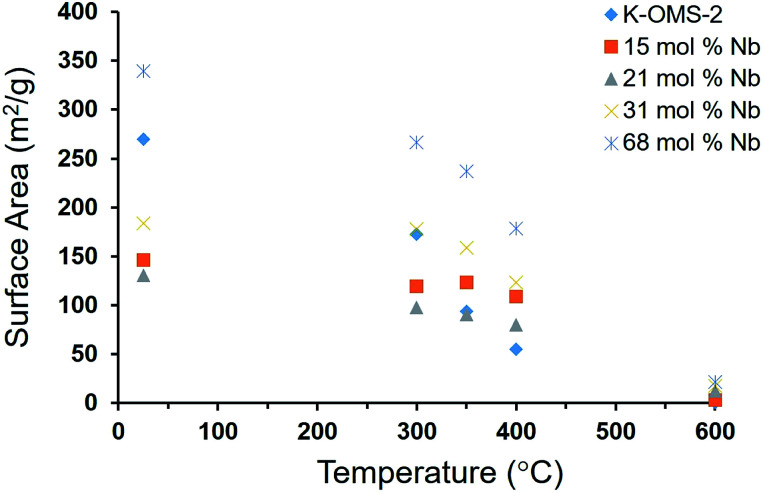
Particle growth and sintering of K-OMS-2 and Nb-OMS-2 materials.

Powder X-ray diffraction (PXRD) was used to determine bulk sample identification and phase purity of the product, and to determine the various loadings of niobium. The PXRD studies were performed with a Rigaku Ultima IV diffractometer using Cu Kα (*λ* = 0.15406 nm) radiation. A beam voltage of 40 kV and a beam current of 44 mA were used. The wide-angle PXRD patterns were collected at 0.02° steps over a 2*θ* range of 10−70° with a continuous scan rate of 2.0° min^−1^. The crystallographic phases were identified by comparing the wide-angle PXRD pattern with the Joint Committee on Powder Diffraction Society (JCPDS) database. The cryptomelane structure was retained upon 15% Nb incorporation (Fig. 1). Peak broadening was observed with increasing Nb loading indicating smaller crystallite-size, consistent with the TEM data. For materials, higher than 31% Nb content, an amorphous powder diffraction pattern was obtained. Since the reactions were carried under a range of temperature, it is important to study the structural changes of the materials with temperature. Thus, *in situ* PXRD data were collected using an *in situ* PANalytical X-ray diffractometer under nitrogen and oxygen flow equipped with Cu Kα (*λ* = 1.5405 Å) radiation within a range of 5° ≤ 2*θ* ≤ 70° (step size: 0.02°, counting time: 1 s per step, ¼ angle incident slit, 45 kV, and a current of 40 mA). 15% Nb-OMS-2 and 21% Nb-OMS-2 materials were able to withstand up to 400 °C under the oxidizing environment. At temperatures above 400 °C significant particle growth and sintering was observed for both the materials (see ESI Fig. S1†). The observed peak sharpening was consistent with BET data in Fig. 2. For 31% Nb-OMS-2 and 68% Nb-OMS-2 materials the extents of particle growth and agglomeration was greater compared to the materials with lower Nb content. Amorphous powder diffraction pattern was obtained for both 31% Nb-OMS-2 and 68% Nb-OMS-2 and produced Nb_2_O_5_ crystallites upon heating above 500 °C (see ESI Fig. S1†).

#### BET surface area

Nitrogen (N_2_) physisorption analysis was performed in a Quantachrome Autosorb iQ instrument to study surface area of undoped K-OMS-2 and Nb doped manganese oxide materials. All the samples were degassed at 150 °C for 6 h under vacuum prior to measurement. The nitrogen adsorption experiments were performed at −196 °C. Twenty-six-point adsorption and twenty-point desorption were performed with *P*/*P*_0_ ranging from 0.002 to 0.995. Points from 0.03 to 0.25 were selected for the BET method giving a *R* value > 0.999 for all samples for surface area calculation. Surface areas of four different Nb loadings 15 mol%, 21 mol%, 31 mol%, and 68 mol% were compared to undoped K-OMS-2. Surface areas of all the materials were decreased upon increasing calcination temperature. All of these materials turn into very dense phases going from 400 °C to 600 °C. Data in Table S1† and in the corresponding Fig. 2 below show that increased Nb loadings from 0 to 15 mol% significantly reduced particle growth and sintering from the as made state to materials calcined at 350 °C. While all K-OMS-2 with no Nb showed ∼70% surface area loss in this heat treatment and retention of the OMS-2 framework structure. The corresponding surface area loss for the 15% Nb-OMS-2 material was only around 20%. Although the extent of sintering and particle growth slightly increased for 21% Nb-OMS-2, the 31% Nb-OMS-2 material and the amorphous material derived from 68 mol% Nb showed similar trends. In materials that had higher than 31% Nb content the OMS-2 framework was no longer retained and hence interpreting the particle growth and sintering mechanism in them became challenging.

Both Quench Solid Density Functional Theory (QSDFT) and Barrett–Joyner–Halenda (BJH) methods were employed to calculate the pore volumes of the materials (Table S2†). When BJH pore volumes from desorption branch is considered (Fig. S2†), it decreases from 0.674 cm^3^ g^−1^ to 0.216 cm^3^ g^−1^ for 15% Nb-OMS-2 to 31% Nb-OMS-2. Furthermore, pore volume increases in 68% Nb-OMS-2 to 0.344 cm^3^ g^−1^. The pore diameter decreases from 17.6 nm to 1.6 nm when considering 15% Nb-OMS-2 to 68% Nb-OMS-2 samples. As far as the pore volumes from QSDFT method on the adsorption branch is considered, it decreases from 0.536 cm^3^ g^−1^ to 0.182 cm^3^ g^−1^ for 15% Nb-OMS-2 to 31% Nb-OMS-2. Furthermore, pore volume increases in 68% Nb-OMS-2 to 0.285 cm^3^ g^−1^. The pore diameter decreases from 29.0 nm to 1.0 nm for 15% Nb-OMS-2 to 68% Nb-OMS-2 samples when QSDFT method is utilized. Therefore, both methods indicate the same trend in the change of pore volumes and pore diameters of materials. The material with 21 mol% Nb loading which shows the highest catalytic activity has a monomodal pore size distribution around 20 nm. 15 mol% Nb sample primarily contains macropores and the pore volume is very high. However, for the samples with 31 mol% and 68 mol% Nb loading, the pore size ranges in a wide range and this may due to the collapsed cryptomelane structure as proven from the XRD data.

#### TEM, STEM and HAADF imaging

**Fig. 3:**
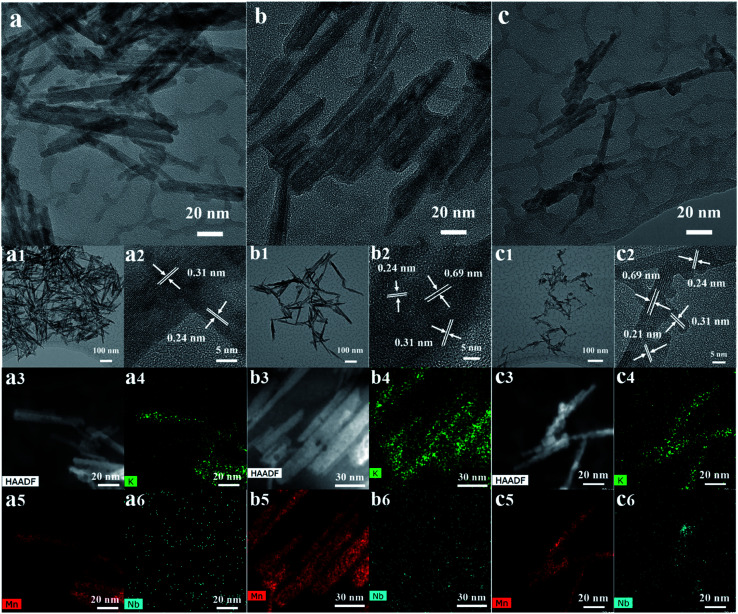
(i) TEM images of (a) 15%-Nb-OMS-2 (b) 21% Nb-OMS-2 (c) 31% Nb-OMS-2 are shown in 20 nm resolution, lower resolution (100 nm) images of corresponding materials are shown in a1, b1, c1 respectively, (ii) a2, b2 and c2 shows selective area diffraction and corresponding *d* spacings, (iii) HAADF images of (a), (b), (c) are shown by a3, b3 and c3 respectively, (iv) elemental mapping for K, Mn and Nb are shown by (a4, a5, a6), (b4, b5, b6) and (c4, c5, c6) respectively.

Fig. 3 below shows TEM images of Nb-doped OMS-2 materials. The scanning transmission electron microscopy (STEM) and transmission electron microscopy (TEM) images with different Nb concentrations are shown above. The TEM images of samples 15% Nb-OMS-2, 21% Nb-OMS-2, and 31% Nb-OMS-2 show typical OMS-2 nanorod morphologies, with average lengths of 121.2 nm, 133.5 nm, and 51.3 nm, and average diameters of 14.2 nm, 11.2 nm, and 5.4 nm and average aspect ratios of 11.2, 9.7, and 9.6, respectively (statistical measurement results for TEM for randomly selected 60 nanorods). Meanwhile, there is detrital Nb species seen in the 31% Nb-OMS-2 material. The increasing Nb loading in OMS-2 leads to smaller crystallite size and the rod like morphology of the OMS-2 materials gradually decrease in terms of aspect ratio. The crystal lattice spacing (*d*) of ∼0.5 nm was observed for the 21 mol% sample but the spacing for the 31 mol% sample appeared to be slightly increased to ∼0.7 nm. After increasing the Nb concentration to 73 mol%, the nanorods completely disappeared. Instead, randomly distributed amorphous mixed Mn, Nb oxides were seen. The selected area electron diffraction (SAED) patterns of both materials show broad diffraction rings, which means the materials have low crystallinity similar to amorphous Mn oxide. The crystal clusters were observed for high resolution TEM, with a *d* spacing value of 0.31 nm, attributed to (310) planes of OMS-2. The STEM mappings show uniform distributions of the elements K, Mn, and Nb.

Elemental mapping of K, Mn and Nb confirmed that while K and Mn track the rod morphology, a small amount of Nb clustering is seen for 31%-Nb-OMS-2. In other materials the Nb distribution appeared to be more uniform.

#### X-ray photoelectron spectroscopy (XPS) analysis

X-ray photoelectron spectra (XPS) characterization of the synthesized materials were done on a PHI model Quantum 2000 spectrometer with scanning ESCA multiprobe (Φ Physical Electronics Industries Inc.), using Al Kα radiation (*l* = 1486.6 eV) as the radiation source. The spectra were recorded in the fixed analyzer transmission mode with pass energies of 187.85 eV and 29.35 eV for recording survey and high resolution spectra, respectively. The powder samples were pressed on a double-sided carbon tape mounted on an Al coupon pinned to a sample stage with a wash and screw then placed in the analysis chamber. Binding energies (BE) were measured for Nb 3d, Mn LMM, Mn 3s, Mn 2p and O 1s regions. The XPS spectra obtained were analyzed and fitted using CasaXPS software (version 2.3.16). Sample charging effects were eliminated by correcting the observed spectra with the C 1 s BE value of 284.8 eV.

**Fig. 4:**
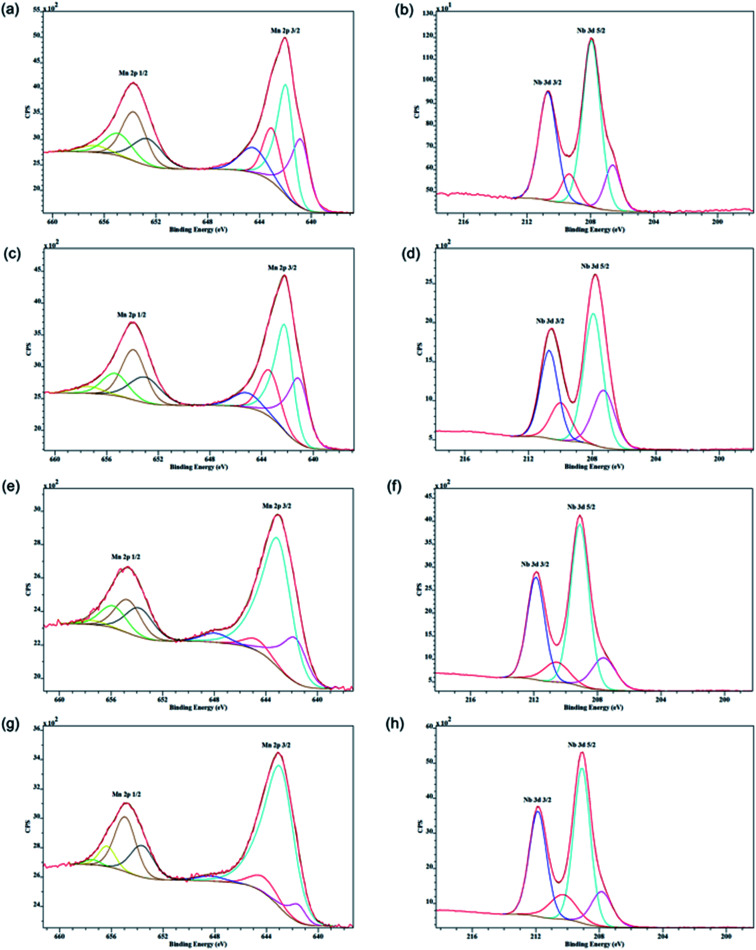
XPS Spectra of Nb-OMS-2 materials: (a and b) 15% Nb-OMS-2, (c and d) 21% Nb-OMS-2, (e and f) 31% Nb-OMS-2, (g and h) 68% Nb-OMS-2.

XPS spectra of the Nb loaded sample are given in Fig. 4. The binding energy of Mn 2p_3/2_ at 641.9 eV of the 15% Nb sample can be ascribed to oxidation states 2+, 3+, and 4+ of manganese.^31^ This suggests the presence of mixed valent manganese species in the material. Both 15% and 21% samples show the binding energy of Nb 3d_5/2_ at 207.9 eV can be ascribed to the 5+ oxidation state of Nb.^32^ However, with higher Nb loading the binding energy of Nb 3d_5/2_ has shifted up to 209.1 eV. The XPS spectra for samples are summarized in Table S3.† Mn 2p_3/2_ binding energies of manganese of all the samples could be ascribed to 2+, 3+, and 4+ states of manganese and the 5+ state of niobium. Very similar XPS data were obtained from other Nb-OMS-2 materials as shown in Fig. 4.

### Catalytic activity

**Fig. 5:**
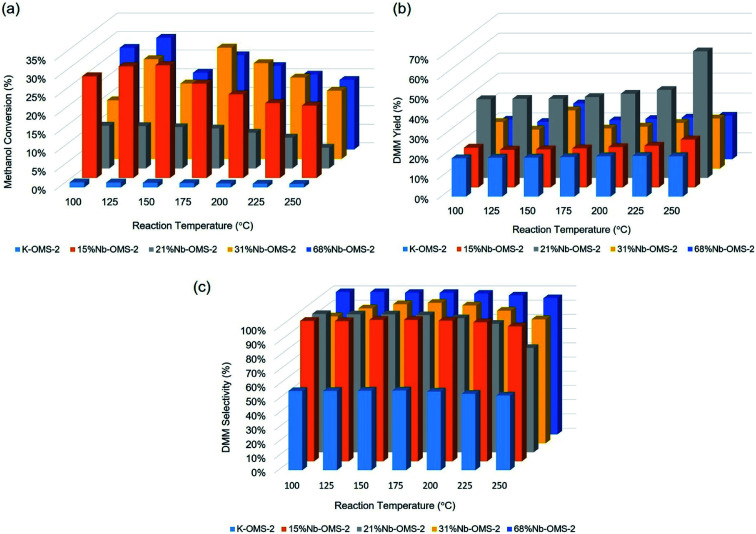
Activity profiles of Nb incorporated OMS-2: (a) methanol conversion *vs.* reaction temperature, (b) DMM yield *vs.* reaction temperature, (c) DMM selectivity *vs.* reaction temperature.

Methanol conversion under given conditions was around 1% for undoped K-OMS-2 for the entire temperature range from room temperature (RT) up to 250 °C. All the Nb incorporated samples show in any case 10% methanol conversion. 15% Nb-OMS-2 sample reached the highest conversion under the given conditions of 30% at 150 °C. 21% Nb-OMS-2 sample shows 12% conversion until 200 °C and then the activity drops. Both 31% Nb-OMS-2 and 68% Nb-OMS-2 samples show similar trend of increasing activity until 125 °C and then maintaining the activity around 20–25% of methanol conversion. Average DMM yield from methanol conversion over the studied samples ranged from 20–25% except for 21% Nb-OMS-2 sample in which the DMM yield was above 40% and reached maximum yield at 65% at 250 °C. Selectivity for DMM was typically around 90–95% for all the Nb incorporated samples. Total oxidation products (CO_2_, H_2_O) were detected at the high end of the temperature range (>200 °C) with methanol conversion typically decreasing in the range, due to either kinetic or physical considerations (*e.g.* coking, quenching of active sites). Normalized activity correlations followed the trend 21% Nb-OMS-2 > 15% Nb-OMS-2 > 31% Nb-OMS-2 > 68% Nb-OMS-2 > K-OMS-2. Interestingly, a fluctuation in methanol conversion was observed around 125–150 °C in most samples, suggesting this to be a catalytically important temperature regime when forming active sites for DMM production (Fig. 5).

## Conclusion

In summary, Lewis acidic and redox innocent Nb(v) can be incorporated into the K-OMS-2 framework by substituting Mn atoms. TEM, STEM, and HAADF imaging of Nb doped OMS-2 materials showed retention of rod like morphologies with subtle changes in *d* spacings and aspect ratios. BET data suggested that upon substitution with Nb, the OMS-2 rods became less susceptible to particle growth and sintering upon heat treatment compared to all Mn K-OMS-2 materials. The cryptomelane rod like morphology of K-OMS-2 is retained even up to 31% substitution of Mn with Nb. At a higher loading of Nb an amorphous solid solution is obtained. Including single crystalline niobium rich Mn oxide materials and all Mn K-OMS-2, this work maps out the structural subtleties of mixed metal oxides of Mn and Nb. Normalized activity correlations followed the trend 21% Nb-OMS-2 > 15% Nb-OMS-2 > 31% Nb-OMS-2 > 68% Nb-OMS-2 > K-OMS-2. Thus having large pores preferably the same size promote the activity of the catalysts. The surface analysis data along with the XRD data thus confirm that not only the incorporation of Nb but also retaining the cryptomelane structure with larger pore volume is promising to achieve a better performance in this study. Interestingly, a fluctuation in methanol conversion was observed around 125–150 °C in most samples, suggesting this to be a catalytically important temperature regime when forming active sites for DMM production.

## Conflicts of interest

There are no conflicts to declare.

## Supplementary Material

RA-009-C9RA04804A-s001
