# Supplementary material for: Niobium-substituted octahedral molecular sieve (OMS-2) materials in selective oxidation of methanol to dimethoxymethane
Source: RSC Adv. 2019 Oct 14;9(56):32665–73. doi: 10.1039/c9ra04804a (PMC9073187; doi:10.1039/c9ra04804a)
Supplement: RA-009-C9RA04804A-s001 [file RA-009-C9RA04804A-s001.pdf]

Electronic Supplementary Material (ESI) for RSC Advances.
This journal is © The Royal Society of Chemistry 2019

Supporting Information

Niobium-substituted Octahedral Molecular Sieve (OMS-2) Materials in Selective Oxidation of Methanol to Dimethoxymethane

Niluka D. Wasalathanthri,[1] Curtis Guild,[1] Quddus A. Nizami,[3] Shanka L. Dissanayake[1], Junkai He,[2] Peter Kerns,[1] Jared Fee,[1] Laura Achola,[1] Dinithi Rathnayake,[1] Chandima Weerakkody,[1] Steven L. Suib,[1-2,*] and Partha Nandi[3,*]

[1] *Department of Chemistry, University of Connecticut, Storrs, Connecticut 06269, United States*

[2] *Institute of Material Science, University of Connecticut, Storrs, Connecticut 06269, United States*

[3] *Corporate Strategic Research, ExxonMobil Research and Engineering Company, Annandale, New Jersey 08801, United States*

Table of Contents: Page No.

Figure 1S:	In situ XRD of Nb-OMS-2	2
Table 1S:	BET Surface area as a function of calcination temperature	2
Figure 2S:	BJH pore size distribution	3
Table 2S:	Comparison of pore volumes and pore diameters	3
Table 3S:	XPS data of Nb-OMS-2 materials	4

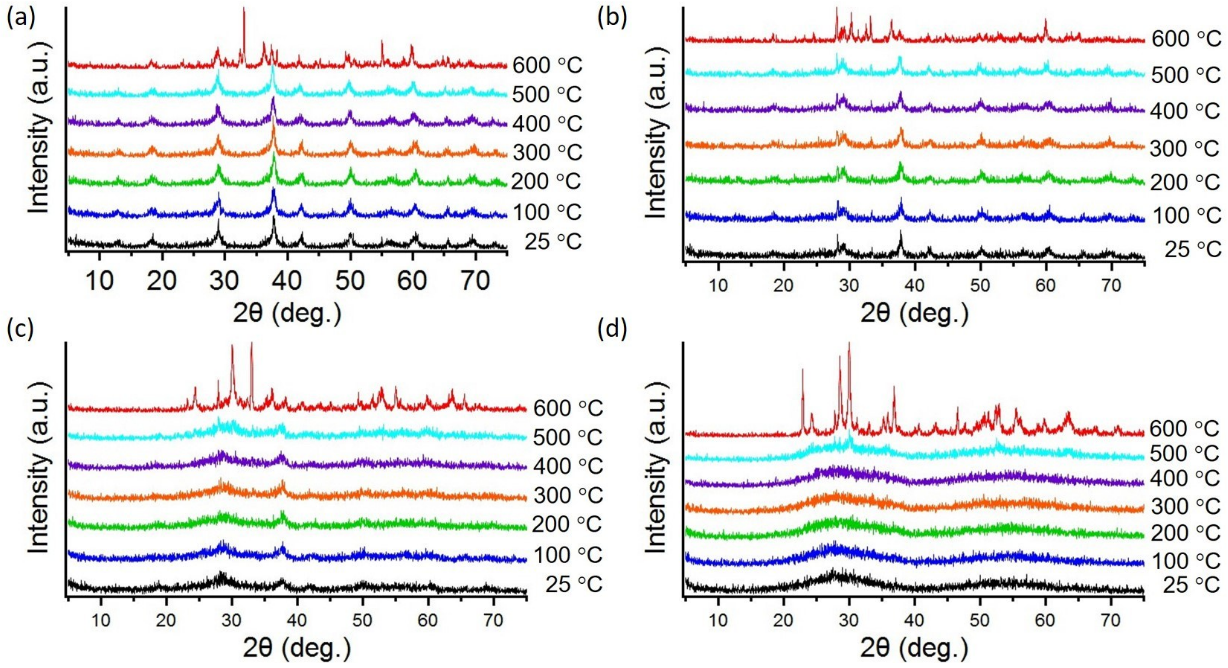

Figure 1S: *In situ* XRD of Nb-OMS-2 heated on an environmental cell under air: (a) 15% Nb-OMS-2 (b) 21% Nb-OMS-2 (c) 31% Nb-OMS-2 (d) 68% Nb-OMS-2

Table 1S: BET Surface area as a function of calcination temperature.

Calcination Temperature (°C)	K-OMS-2 Surface Area (m^2/g)	Nb:Mn (0.15) Surface Area (m^2/g)	Nb:Mn (0.21) Surface Area (m^2/g)	Nb:Mn (0.31) Surface Area (m^2/g)	Nb:Mn (0.68) Surface Area (m^2/g)
As synthesized	269	147	131	184	339
300	173	120	98	178	266
350	94	124	90	159	237
400	55	109	80	123	178
600	0.6	3	13	18	22

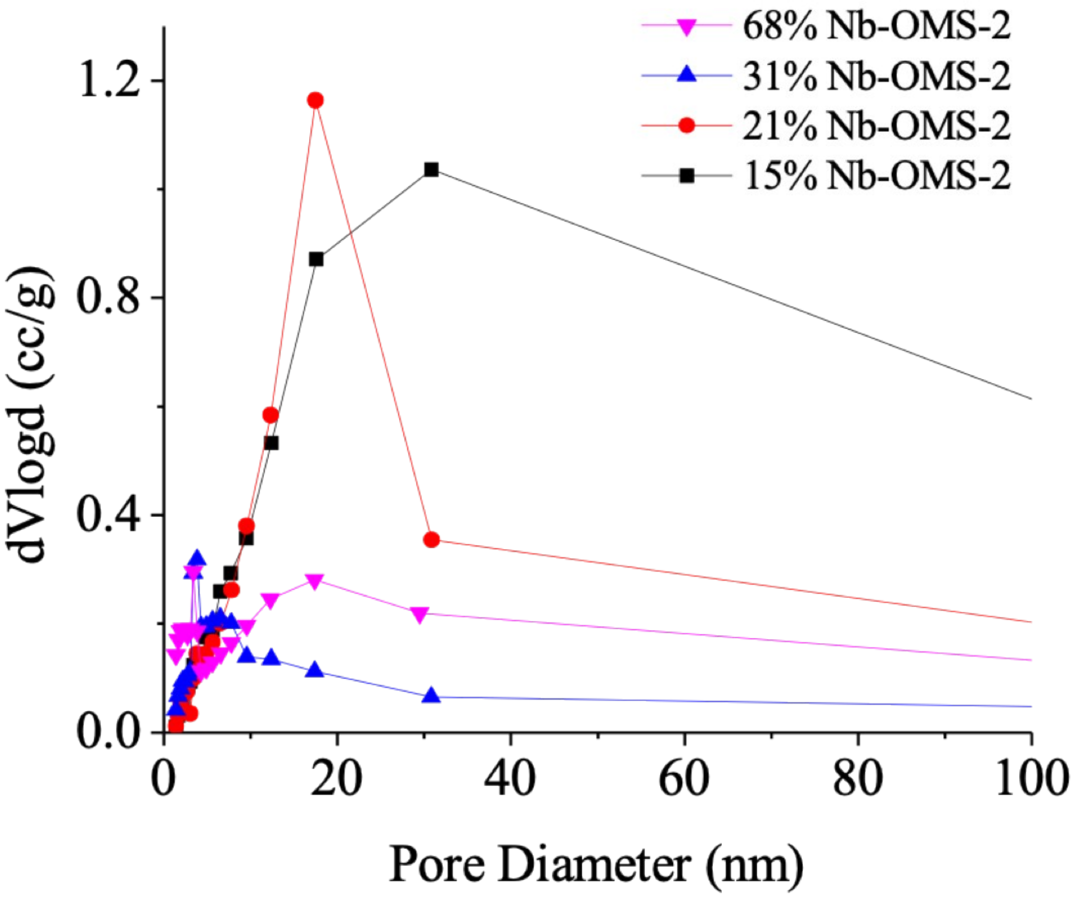

Figure 2S: BJH pore size distribution.

Table 2S: Comparison of pore volumes and pore diameters calculated using BJH method and QSDFT method.

Mol% Nb loading	BJH Method		QSDFT Method	
	Pore Volume (cc/g)	Pore Diameter (nm)	Pore Volume (cc/g)	Pore Diameter (nm)
15	0.674	17.6	0.536	29.0
21	0.518	17.5	0.457	29.0
31	0.216	3.4	0.182	1.3
68	0.344	1.6	0.285	1.0

Table 3S: XPS data of Nb-OMS-2 materials.

Mol% Nb loading	$Mn^{2p3/2}$	$Mn^{2p1/2}$	$Nb^{3d5/2}$	$Nb^{3d3/2}$
15	641.9	653.7	207.9	210.6
21	642.2	653.9	207.9	209.7
31	643.1	654.7	209.1	211.8
68	642.9	654.9	209.0	211.8
